# VNAR: shark single-domain antibodies for the new era of medical biotechnology

**DOI:** 10.3389/fimmu.2025.1716916

**Published:** 2026-02-02

**Authors:** Richard A. Olivares-Olivares, Angélica R. Bravo, Carlos Garrido-Soto, Jonatan J. Carvajal, Augusto Manubens, Mariella Rivas, Carlos Bustamente, Angello Retamal-Díaz, Alexis M. Kalergis, Margarita K. Lay

**Affiliations:** 1 Departamento de Biotecnología, Facultad de Ciencias del Mar y Recursos Biológicos, Universidad de Antofagasta, Antofagasta, Chile; 2 Centro de Investigación en Inmunología y Biotecnología Biomédica de Antofagasta (CIIBBA), Hospital Clínico Universidad de Antofagasta, Universidad de Antofagasta, Antofagasta, Chile; 3 Laboratorio de Microbiología Celular y Fotodinámica, Centro de Ciencias Médicas Aplicadas, Facultad de Ciencias de la Salud y Medicina, Universidad Central de Chile, Santiago, Chile; 4 CIENBIO, Santiago, Chile; 5 Laboratorio de Biología Pesquera, Facultad de Ciencias del Mar y Recursos Biológicos, CHALLWA, Instituto Alexander von Humboldt de Ciencias Naturales, Universidad de Antofagasta, Antofagasta, Chile; 6 Instituto Milenio de Inmunología e Inmunoterapia, Universidad de Antofagasta, Antofagasta, Chile; 7 Instituto Milenio de Inmunología e Inmunoterapia, Facultad de Ciencias Biológicas, Pontificia Universidad Católica de Chile, Santiago, Chile; 8 Departamento de Endocrinología, Facultad de Medicina, Pontificia Universidad Católica de Chile, Santiago, Chile

**Keywords:** biomedical applications, display technologies, IgNAR, medical biotechnologies, shark antibodies, single-domain antibodies, VNAR

## Abstract

Shark-derived single-domain antibodies, known as VNARs, represent unique and advanced tools in medical biotechnology. Recognized for their small size, simple structure, and exceptional stability, VNARs can access cryptic epitopes that are inaccessible to traditional antibodies, making them valuable tools for next-generation diagnostic and therapeutic applications. Additionally, their evolutionary origin and structural diversity provide resistance to extreme pH, temperature, and proteolytic environments, making them especially suitable for demanding biomedical settings such as ocular and intestinal applications. Recent progress highlights their growing clinical potential: VNAR-based CAR-T cells targeting PD-L1 demonstrated strong anti-tumor effects in preclinical assays, with VNAR-B2 successfully blocking PD-L1/PD-1 interactions and reducing tumor growth in mouse models. Meanwhile, the TXB2 VNAR platform allows efficient, non-invasive transport of biologics across the blood-brain barrier. These developments emphasize VNARs’ advantages over traditional antibodies and even camelid VHHs in targeting difficult-to-reach sites and environments. Additionally, commercial development in VNAR technologies is advancing, with companies like Elasmogen using its soloMER™ platform to develop shark-derived, humanized single-domain antibodies for challenging therapeutic environments. This review consolidates emerging insights into VNAR structural biology, display technologies (phage, ribosome, yeast, and bacterial), and library engineering strategies, emphasizing their growing role in immunodiagnostics, infectious disease detection, targeted therapies, and barrier-crossing biologics. It addresses key translational challenges such as humanization and half-life extension, which are crucial for clinical application, ultimately highlighting the transformative potential of VNARs in bridging vital gaps in modern medicine.

## 1 Introduction

**Figure 1:**
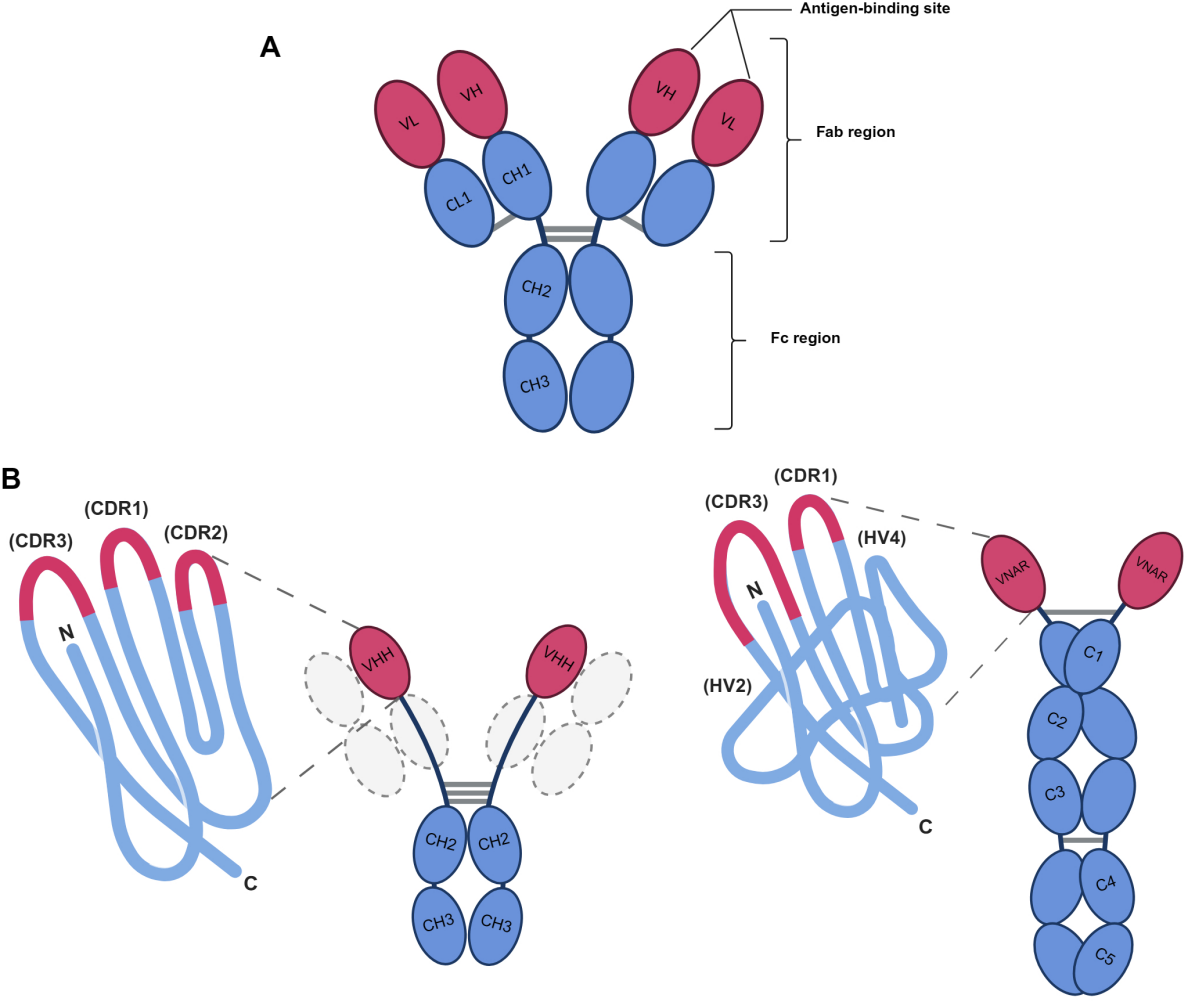
Comparative structure of conventional antibodies (IgG) and single-domain antibodies (VHH and VNAR). **(A)** Schematic illustration of a conventional IgG antibody. The Fc region (crystallizable fragment), composed of the constant CH2 and CH3 domains of the heavy chains, mediates the effector functions of the antibody, including system complement activation and interaction with Fc receptors on immune cells such as macrophages, neutrophils, and NK cells, facilitating phagocytosis and pathogen clearance. **(B)** Structural comparison between sdAbs from camelids and sharks. Although both sdAbs lack light chains, they are structurally distinct, particularly in the organization and diversity of their CDR/HV regions of the variable domains. Created with BioRender.com.

Antibodies (Abs) or immunoglobulins (Igs) are essential proteins in the body’s defense against pathogens, including bacteria, viruses, fungi, and toxins. Their functional versatility — ranging from antigen neutralization to immunological memory and therapeutic applications — has revolutionized immunology and modern medicine; in this review, the abbreviation Abs will be used to refer to them. Structurally, conventional Abs adopt a “Y” shape, composed of two heavy chains (H) and two light chains (L), with variable heavy (VH) and light (VL) regions that determine antigenic specificity and constant heavy (CH) and light (CL) regions, respectively, which define their class and the function of their effects (1). These chains are assembled by disulfide bonds, forming two key fragments: the antigen-binding fragments (Fab), which binds antigens; and the crystallizable fragment (Fc), which mediates biological effects, such as complement activation (**Figure 1A**). Despite their efficacy, conventional Abs have limitations including: their large size ~150 kilo Daltons (kDa) that restricts tissue penetration: and their structural complexity, which makes recombinant production difficult. This prompted the search for smaller, more stable formats, leading to the discovery of the single-domain antibodies (sdAbs) (2) (**Figure 1B**).

In the 1990s, a novel class of sdAbs was discovered in cartilaginous fishes and camelids. Unlike classical Abs, these molecules feature a simplified structure, lacking light chains. Specifically, they are composed exclusively of a variable heavy chain domain, called the variable heavy chain domain of heavy-chain-only antibodies (VHH) in camelids or the variable new antigen receptor domain (VNAR) in sharks (3–5), with a molecular weight of only 12–15 kDa. In camelids (*Lama glama*, *Vicugna pacos*), these are known as heavy chain antibodies (HCAbs), whereas in sharks (e.g., *Ginglymostoma cirratum*), these are known as novel antigen receptors (IgNARs). VHH domains, recombinantly produced for clinical applications, have been marketed as Nanobodies^®^ (Ablynx/Sanofi), highlighting their therapeutic potential (6).

Although VHHs dominate the therapeutic landscape, VNARs have attracted attention for their applicability diagnostic applications and potential in oncology and infectious diseases. This review synthesizes the current knowledge on VNARs, addressing: (1) their structural and physicochemical properties, (2) their production methods (immunized sharks, synthetic libraries), (3) therapeutic and diagnostic applications, and (4) biotechnological platforms for their development. The objective is to highlight the emerging role of these unique shark antibodies in biomedical innovation and the remaining challenges for their clinical implementation (3, 7, 8). Unlike VHHs, VNARs possess a unique structural framework (types I–IV) defined by the arrangement of their disulfide bridges, as well as an elongated complementarity-determining regions (CDR) 3 that serves as an antigen-binding arm. Furthermore, their low abundance in sharks (<1% of the repertoire) suggests a functional specialization that has yet to be fully explored.

## 2 Origins, structural characteristics, and biomedical applications of VNARs

### 2.1 Evolutionary development of the adaptive immune system in cartilaginous fishes

**Figure 2:**
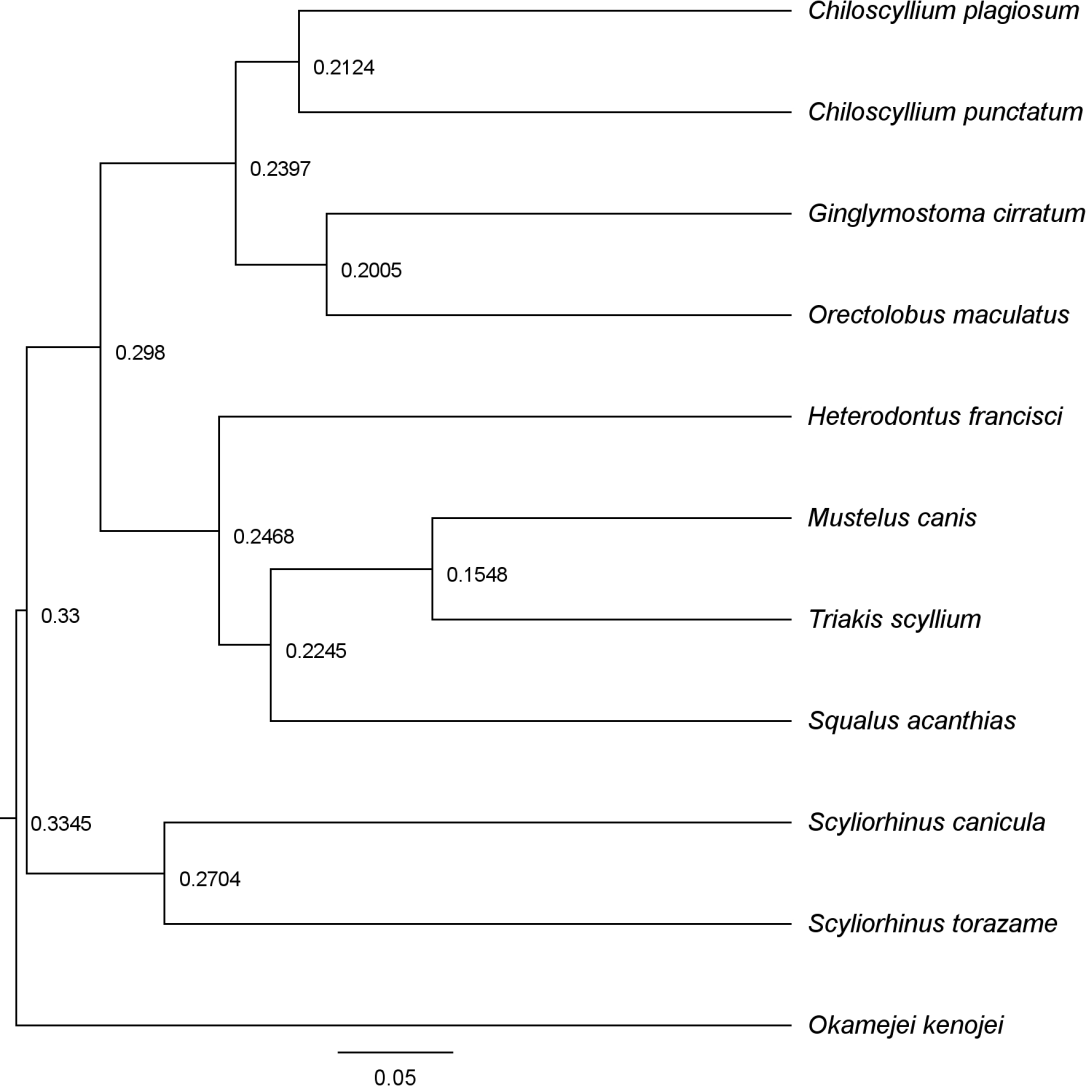
UPGMA dendrogram of 11 VNAR domains from different cartilaginous fishes. VNAR amino acid sequences from sharks and a ray were analyzed in Geneious Prime using global pairwise alignment with end gaps and the BLOSUM62 substitution matrix. Distances between pairwise sequences (proportion of amino acid differences) were estimated using the Jukes-Cantor model. The horizontal scale bar represents the expected proportion of amino acid substitutions per site. The tree highlights species pairs with highly conserved VNAR domains, providing a framework for the rational design of universal versus species-specific primers for VNAR domain amplification in other elasmobranch species.

Cartilaginous fishes (*Chondrichthyes*), which include sharks, rays, and chimaeras, represent the oldest living vertebrate lineage that retains a functional adaptive immune system. This group diverged from bony vertebrates approximately 450 million years ago, retaining primitive traits that offer a unique window into understanding the evolution of humoral immunity and antigen recognition mechanisms in early vertebrates (4, 9) (**Figure 2**). Phylogenetic analysis of VNAR amino acid (aa) sequences reveals a substantial intraspecific and intragenic similarity, with closely related species tending to cluster together with low genetic distances. This high degree of sequence conservation within groups reflects evolutionary pressures to maintain VNAR function. It also provides a molecular basis for designing universal or species-specific primers for VNAR amplification in novel species.

Evolutionarily, the immunoglobulin IgNAR — from which VNAR domains are derived — is postulated to have arisen because of a duplication and divergence event in ancestral immunoglobulin heavy chains gene. This process was likely a selective adaptation to the pressure exerted by complex and persistent marine pathogens, such as enveloped viruses, saline-resistant bacteria, and multicellular parasites. The fact that IgNAR coexists with other Ig isotypes such as IgM and IgW suggests that this molecule fulfills specialized functions, possibly optimized for the recognition of repetitive antigens or for functioning in marine mucosal environments (10).

The immune system of these fishes is estimated to have emerged approximately 450 million years ago (11). This system comprises components such as the major histocompatibility complex (MHC), T cell receptors (TCR), and three classes of Ig: IgM, IgW, and the unique IgNAR (4, 9, 12, 13). The immune system of cartilaginous fishes, unlike that of mammals, lacks bone marrow and lymph nodes. In this sense, these animals possess the gut-associated lymphoid tissue (GALT), the thymus (necessary for the maturation of T lymphocytes) and unique lymphomyeloid tissues such as the epigonal and Leydig organs (sites of B cell lymphopoiesis), with the spleen being the primary immune organ (12, 14). However, secretory Ig transcripts have also been detected in the kidney and liver of these fishes (15).

These organisms display all the fundamental mechanisms of adaptive immunity, including recombination-activating gene (RAG), variable (V) – diversity (D) – joining (J) rearrangement (V(D)J recombination), somatic hypermutation, and clonal selection (12, 16). Among the most notable components of the immune system are IgNARs, which are composed of two homodimeric heavy chains and completely lack light chains. The resulting VNAR possesses hypervariable and flexible CDR3 domains. These features grant them attractive biomedical properties, since they recognize epitopes inaccessible to conventional Abs, have high thermostability, resistance to extreme pH and high tissue penetration due to their small size (17).

IgNAR production is initiated by specialized B cells, located mainly in the spleen, the epigonal organ, the Leydig organ (if present), and the GALT. These B cells undergo somatic recombination of immunoglobulin genes via RAG1/2 mechanism, thereby generating diversity in the VNAR repertoire (16). However, unlike conventional Abs, VNARs have unique structures with highly diversified CDRs, especially CDR3, which can reach unusual lengths (>25 residues aa), expanding their binding capacity to cryptic epitopes (16).

IgNAR synthesis in sharks represents a highly specialized process within the ontogenetic development of the adaptive immune system of cartilaginous fishes (4, 12). This process begins with the generation of genetic diversity through V(D)J somatic recombination in lymphoid progenitor cells. At this stage, the variable V(D)J segments undergo precise genomic rearrangement, giving rise to the functional transcript of the VNAR domain, corresponding to the variable region of the IgNAR Abs.

Somatic recombination in IgNARs is remarkable complex, typically involving one V segment, three D segments, and one J segment. This intricate recombination process generates exceptionally long and structurally diverse CDR3 hypervariable regions (5), that harbor non-canonical cysteine residues. These cysteine residues facilitate the formation of intra-CDR disulfide bridges, thereby enhancing the structural stability of the VNAR domain. Although IgNARs lack light chains and consequently cannot exploit the inter-chain combinatorial diversity characteristic of conventional Abs, they compensate for this limitation through several mechanisms. These include additional recombination events, the utilization of multiple D segments, and terminal deoxynucleotidyl transferase (TdT)-mediated incorporation of N and P nucleotides during gene segment joining. The IgNAR gene locus is structured as independent cassettes, each containing the V, D, and J segments, as well as the constant heavy exons. Species such as *Ginglymostoma cirratum* (nurse shark) have been identified to have up to four functional IgNAR loci, increasing the diversity of the repertoire (18).

Following recombination, IgNAR-expressing B cells migrate to peripheral lymphoid organs, such as the spleen and mucosal-associated lymphoid tissues (MALT). At these sites, clonal selection occurs, eliminating autoreactive lymphocytes and favoring those with functional affinity for antigens (12, 19). Since cartilaginous fishes lack lymph nodes, these tissues play analogous roles in B cell maturation and expansion.

During active immune responses, B cells expressing secretory IgNAR undergo somatic hypermutation (SHM) mediated by the enzyme activation-induced cytidine deaminase (AID), which introduces point mutations in the variable regions. This process enables affinity maturation and further diversification of the immunological repertoire. The constant regions constant heavy CH2–CH5 are transcribed and translated along with the variable region in the rough endoplasmic reticulum, where the protein is folded, glycosylated, and assembled before secretion as a functional Ab (15, 20, 21).

Finally, activated B cells undergo clonal expansion and differentiate into IgNAR-secreting effector cells, capable of recognizing a wide range of antigens, including hidden epitopes inaccessible to other Ig types (18, 22). This process yields thermostable Abs, which are essential for immune function in sharks and other cartilaginous fishes.

The long-lasting presence of this system for over 400 million years demonstrates its evolutionary success and supports the idea that VNARs perform immunological functions that other Igs cannot replace.

### 2.2 Structural characteristics and folding of VNARs

VNARs are the variable domains of the immunoglobulin IgNAR, unique to cartilaginous fishes. These structures represent the most reduced and primitive form of functional Ig domains known in vertebrates, with a molecular mass of approximately 12–15 kDa (~110 aa) (21). Notwithstanding their compact size and absence of light chains, VNARs exhibit high-affinity binding to specific antigenic epitopes due to their highly specialized structural organization (19).

Abs are characterized by the absence of the CH1 domain resulting from alternative splicing, which prevents light chain pairing. Whereas camelids typically harbor two constant domains, sharks can harbor up to five constant domains (5, 19). This gives them a greater advantage over these sdAbs.

From a structural perspective, VNARs maintain the Ig-like fold, comprising a hydrophobic core stabilized by two antiparallel β-sheets, a feature typical of the “β-sandwich” fold characteristic of Ig-like domains. This structural core is stabilized by a disulfide bridge between two conserved cysteines, located at the ends of the β-chains (10, 23). However, VNARs differ significantly from the VH variable domains of conventional Abs in several key aspects, such as:

- They lack the classic CDR2 present in mammalian Abs. An additional hypervariable region called the heavy chains variable (HV) domain 2 functionally compensates for this region and may participate in the interaction with the antigen (24).
- They present two main functional hypervariable loops CDR1 and CDR3. The latter being highly diverse in both sequence and length (up to 40 residues), providing a large contact surface with the antigen (25). This property enables VNARs to bind to epitopes that are inaccessible to conventional Abs and camelid VHHs (26).

This unique configuration, which combines high stability with extended CDR3 loops to access sterically restricted sites, makes VNARs an ideal platform for therapeutic development, particularly when optimized through the display technologies discussed in the following sections.

### 2.3 Structural classification: types I, II, III and IV

**Figure 3:**
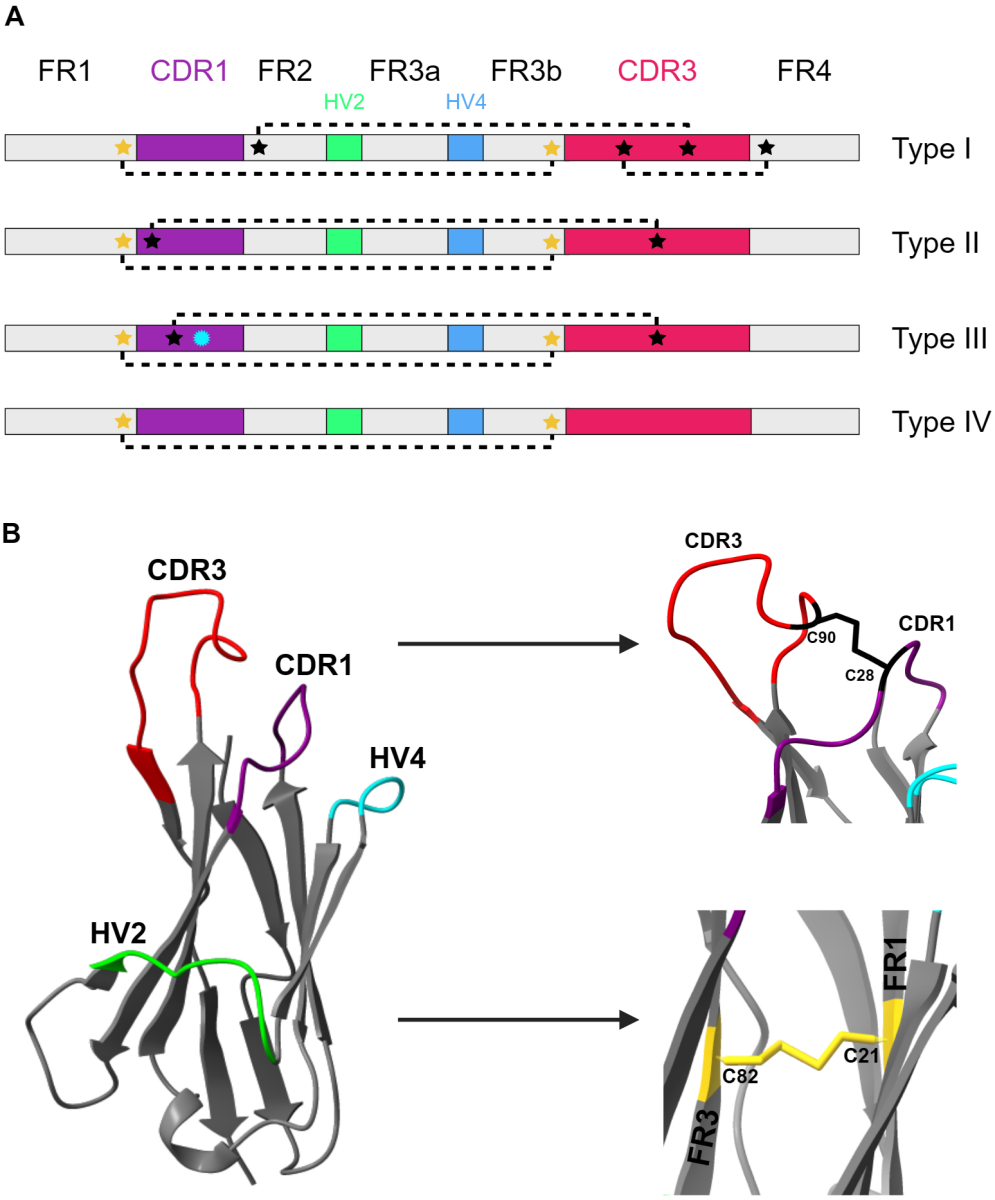
Classification of VNAR isotypes based on cysteine patterns. **(A)** Structural organization and cysteine patterns in the VNAR domains of elasmobranchs. The linear diagrams represent the four classic VNAR types (I–IV), showing the distribution of the structural framework regions (FR1–FR4, gray), CDR1 (purple), CDR3 (magenta), and the hypervariable regions HV2 (green) and HV4 cyan). Canonical cysteines are indicated by yellow stars, non-canonical cysteines by black stars, and disulfide bonds by dashed lines. In type III VNAR, a cyan starburst denotes a conserved tryptophan residue within CDR1. Each type of VNAR exhibits characteristic disulfide bond patterns between its cysteines, reflecting its high structural variability. **(B)** Crystal structure of a type II VNAR from *Orectolobus maculatus* (PDB ID: 2YWZ) (167). Structural regions are color-coded as follows: CDR1 (purple); HV2 (green); HV4 (cyan); CDR3 (red); and FRs (gray). The non-canonical disulfide bond connecting CDR1 and CDR3 is depicted in black. This interaction constrains the CDR3 loop in an extended conformation, facilitating access to cryptic epitopes. The canonical disulfide bond connecting FR1 and FR3 is shown in yellow, contributing to the stability of the hydrophobic core. Figure generated using UCSF ChimeraX version 1.10.1 (168), and BioRender.com.

Due to the great diversity of VNARs, it has been necessary to classify them based on characteristics shared by most VNARs. The “classic” classification comprises four types of VNARs, based on the number and position of non-canonical cysteine residues in CDRs and framework regions (FRs). In addition, these four types of VNARs possess a pair of canonical cysteines at positions 21 (FR1) and 82 (FR3) in their amino acid sequence (25, 27) (**Figures 3A, B**).

#### 2.3.1 Type I

Type I VNAR has four additional non-canonical cysteines, two in the CDR3 region and one in FR2 and FR4, respectively. This arrangement causes CDR3 to adopt a bent, protruding conformation by forming two disulfide bonds with the FRs, generating a flat binding surface that still allows ample access to pockets and grooves (22, 27).

#### 2.3.2 Type II

Type II VNAR has an additional pair of non-canonical cysteines in CDR1 and CDR3. This arrangement favors an extended and protrusive CDR3 by forming a disulfide bond with CDR1, thereby facilitating access to hard-to-reach targets, similar to type I VNARs (22, 25).

#### 2.3.3 Type III

Type III VNAR is like type II. It contains a cysteine in the CDR1 region, next to a highly conserved tryptophan (Trp) residue, and an additional cysteine in CDR3 (28).

#### 2.3.4 Type IV

Type IV VNARs represent the simplest architecture, as they retain only the two canonical cysteine residues FR1–FR3 and do not have additional non-canonical cysteine residues (27). The arrangement of cysteines is not the only factor in Ab-antigen interactions; residues beyond CDR1 and CDR3 may also make contact with the antigen (22). Current IgNAR classifications are based primarily on the location of non-canonical cysteines in the variable region. However, due to the high diversity of these variants, many do not fit into the classical classification (25, 29). Therefore, a new classification based on nucleotide variations in the C1 domain has been proposed, given its uniqueness compared to other constant domains, its role in the geometry of the VNAR (and thus in its ability to interact with the antigen), and its polymorphic nature (29, 30).

### 2.4 Stability and physicochemical properties

One of the most distinctive properties of VNARs is their extraordinary structural stability. This attribute is critical for biomedical applications, which require extreme conditions, such as oral formulations, enzyme therapies, or treatments in inflammatory tissues where pH and temperature vary widely (31).

Biophysical studies have shown that any VNAR: maintains its native structure and functionality above 65-80 °C without irreversible denaturation (32); is resistant to extreme pH (both acidic and basic) and retains its antigen-binding capacity even in the presence of detergents or organic solvents (17); exhibits high solubility without the need for post-translational modifications, avoiding the aggregation that limits the use of conventional antibodies (17); is resistant to proteolysis, making it suitable for oral or local formulations where other antibodies would rapidly degrade (17, 33); exhibits a high antigen-binding capacity (34, 35); and possesses unique HV loops that can bind to antigens independently, sometimes generating bispecificity and increasing its versatility to target various epitopes.

These properties derive in part from their compact size, their architecture stabilized by multiple disulfide bonds, and the absence of exposed flexible or hydrophobic domains. A key challenge associated with VNARs is their rapid renal clearance, due to their low molecular weight (36), leading to a short serum half-life. This limitation has driven the development of half-life extension strategies.

Fusion to human serum albumin or to albumin-binding VNARs such as E06 increases the hydrodynamic size and couples VNARs to the long intrinsic persistence of albumin, extending the circulation time of fused partners from hours to days or weeks in preclinical species and yielding predicted terminal half-lives of ~19 days in humans, comparable to native albumin (17, 27). VNAR-Fc formats similarly exploit receptor Fc neonatal (FcRn)-mediated recycling, providing IgG-like pharmacokinetics with multi-day systemic exposure instead of rapid renal clearance, as shown for anti-ICOSL VNAR-Fc and transferrin receptor 1-targeted TXB2-hFc brain-shuttle constructs (36, 37).

In parallel, PEGylation of Abs fragments increases hydrodynamic radius and reduces glomerular filtration. Furthermore, PEG-conjugated Fab molecules, such as certolizumab pegol, achieve elimination half-lives of ~14 days, several-fold longer than the unmodified fragments, illustrating how covalent PEG shields lower molecular weight scaffolds from rapid renal clearance (38, 39).

## 3 Display technologies for VNAR selection: established and emerging approaches

Across display platforms, diversity is encoded at the level of individual cell transformants or as purely *in vitro* deoxyribonucleic acid (DNA)/messenger ribonucleic acid (mRNA) repertoires. In the former case, each clone corresponds to a single VNAR variant propagated in a microbial or yeast host, while in cell-free systems, the selection acts directly on large populations of translated sequences without the constraints of transformation efficiency or host viability (40–42). This combination of clone-based and sequence-based formats increases the probability of recovering rare, high-affinity, or otherwise developable VNAR variants from immune, naïve or semi-synthetic repertoires.

### 3.1 Phage display

Phage display, typically using filamentous bacteriophages such as M13, was the first and remains the most widely used technique for isolating shark VNARs, both from immune and synthetic/semi-synthetic libraries. On this platform, VNAR-encoding sequences are fused to a phage coat protein (commonly pIII), allowing each phage to display a unique VNAR on its surface while carrying the corresponding genetic information internally, thereby preserving the genotype-phenotype link (43). The phage population is subjected to biopanning against the target antigen: phages binding to the immobilized antigen are recovered, amplified in *Escherichia coli* (*E. coli*), and reselected through several rounds, typically 3–5 cycles, to enrich high-affinity binders (44).

A significant strength of phage display is its ability to handle extensive libraries, ~10^9^–10¹^0^ unique variants, and up to ~10¹¹ in some cases (45, 46), and its compatibility with a wide range of selection formats (plate-based, solution-phase, whole cells, or tissues). This scale and versatility have enabled to the isolation of VNARs with affinities in the low-nanomolar (nM) range, and even sub-nM clones under stringent panning conditions (e.g., low antigen concentration, competition with ligands) (47, 48). The oxidative periplasmic folding environment of *E. coli* during phage assembly supports correct disulfide bond formation, which is particularly important for VNARs’ structural integrity (49).

Phage display has been successfully applied to various VNAR library types. Immune VNAR libraries—like those of sharks of the order *Orectolobiformes* — have yielded highly stable and specific binders to protein antigens (43). Semi-synthetic VNAR libraries, constructed on shark-derived frameworks and diversified *in vitro*, have also been displayed on phages to isolate binders against bacterial toxins without the need for animal immunization (50). In these contexts, the platform’s robustness and its ability to propagate bacteria make it attractive for both research and preclinical development. Affinity maturation can be readily implemented within phage display workflows. Techniques such as error-prone polymerase chain reaction (epPCR), chain shuffling, or targeted CDR mutagenesis allow the generation of second-generation libraries from lead clones, followed by iterative panning to select for an improved binding (19). This iterative process has achieved improvements from nM to sub-nM dissociation constants (K_d_), reaching values as low as 1.0×10^–10^ M (51), demonstrating the platform’s adaptability to laboratory-driven molecular evolution.

However, limitations remain. Library construction requires cloning into phagemid vectors, transformation, and rescue with helper phages, which can be time-consuming and introduce selection biases, and display levels per phage are relatively low (a few copies per particle) (52). Nonspecific binding to complex targets (e.g., whole cells) can further complicate selections, and specific VNAR sequences may be deleterious to the phage or host bacteria, leading to their loss during amplification (53). Despite these drawbacks, the technique’s track record across both camelid VHHs and shark VNARs, combined with its scalability, low cost, and proven compatibility with affinity maturation, ensures it remains a central platform in VNAR discovery (49).

### 3.2 Ribosome display

Ribosome display is an entirely *in vitro* selection technology in which the nascent polypeptide remains physically linked to the ribosome and its encoding mRNA, forming a stable peptide-ribosome-mRNA (PRM) complex (41, 54). By omitting a stop codon in the mRNA, translation stalls and the incomplete polypeptide remain tethered to the ribosome, which can then be incubated with an immobilized target antigen. PRM complexes that bind the target are recovered, dissociated, and the bound mRNA is reverse transcribed into complementary DNA (cDNA) for amplification and subsequent selection rounds (54). Because the process occurs entirely *in vitro*, it bypasses transformation and host cell growth, enabling the screening of extremely large libraries — typically 10¹²–10¹^4^ unique sequences — far exceeding the diversity achievable in cell-based systems such as phage display (10^9^–10¹^0^) or yeast display (10^6^–10^8^) (55, 56). This vast diversity increases the likelihood of isolating rare high-affinity or unique binders. Another significant advantage is speed: multiple selection rounds can be completed within 3–5 days, compared to one or two weeks for phage display (55). A compelling feature is the ability to introduce mutations between selection rounds. Since the recovered genetic material is in DNA form, epPCR, DNA shuffling, or site-directed mutagenesis can be applied to generate diversity for *in vitro* affinity maturation (57, 58). This has been shown to improve affinities by orders of magnitude within a few iterative cycles, effectively simulating somatic hypermutation in a test tube.

Ribosome display is also free from *in vivo* selection biases, sequences that might be toxic to bacterial or yeast hosts — such as specific VNAR frameworks — can still be retained and evolved *in vitro* (42, 56). This has enabled the selection of functional camelid VHHs and shark VNARs against diverse targets, including toxins and membrane proteins (59, 60).

However, the method has limitations. The PRM complex is inherently unstable and requires optimized conditions to prevent dissociation, often relying on high magnesium (Mg²^+^) concentrations and low temperatures (41, 61). Furthermore, because folding occurs co-translationally without cellular chaperones, proteins with multiple disulfide bonds (such as VNARs) may misfold in the reducing environment of *E. coli* lysates (55). This can be mitigated by using oxidizing systems, such as rabbit reticulocyte lysates or engineered bacterial extracts (62). Additionally, ribosome display lacks post-translational modifications, which may affect proteins that require glycosylation or specific folding assistance.

Despite these challenges, ribosome display has proven to be a high-diversity, fast, and evolution-friendly platform for binder discovery. When optimized, it can produce binders with affinities in the low nM to picomolar (pM) range, making it a competitive alternative or complement to cell-based display systems (25, 57, 58).

### 3.3 Yeast display

Yeast surface display is a eukaryotic cell-based selection platform in which Abs fragments, such as VNARs, are genetically fused to an outer cell wall protein — most commonly the *Saccharomyces cerevisiae* Aga2p protein — enabling their presentation on the yeast cell surface while retaining the encoding plasmid internally (63, 64). This genotype-phenotype linkage enables direct interrogation of binding properties by flow cytometry (FACS), allowing simultaneous evaluation of affinity, specificity, and expression levels in a single experiment (65).

A major advantage of yeast display over prokaryotic systems is its eukaryotic folding and secretory pathway, which can correctly process complex proteins containing disulfide bonds (66). This is particularly relevant for shark VNARs, whose frameworks often contain multiple cysteine residues that form additional disulfide bonds beyond the canonical Ig fold (67). Unlike phage or ribosome display, yeast display enables real-time quantitative selection by titrating fluorescently labeled antigens and sorting the highest-affinity binders using FACS (65, 68). It also allows direct counter-selection against off-targets or undesired epitopes during the same sorting process.

Library sizes in yeast display are generally smaller (10^6^–10^8^ transformants) than in phage or ribosome display due to lower transformation efficiency (64, 66). However, this is offset by the ability to rapidly enrich for high-affinity clones in just 2–3 days of sorting once the library is established (65). Affinity maturation is straightforward: yeast populations can be mutagenized and resorted under increasing stringency (e.g., lowering antigen concentration or using competitive binding), enabling the selection of sub-nM binders (68, 69).

In the context of VNARs, yeast display has been successfully employed with semi-synthetic libraries to isolate binders against challenging targets without the need for shark immunization. In addition, the following have been reported: the use of a semi-synthetic VNAR library centered on CDR3, visualized in yeast (49, 70, 71); and the use of dual visualization platforms on the surface of yeast, which has allowed simultaneous visualization of VNARs and epitope tags, thus facilitating the high-throughput characterization of the binding of these molecules to multiple antigens (72). These studies highlight the adaptability of yeast display for both discovery and detailed functional profiling of VNARs.

Limitations of yeast display include slower growth rates compared to *E. coli*, potential size constraints on the displayed protein, and lower maximum library diversity. However, its ability to provide direct quantitative affinity measurements, perform multi-parameter selections, and fold cysteine-rich proteins efficiently makes it a valuable tool for VNAR engineering, particularly when precision in epitope targeting or functional assays are required (47, 49, 63, 70).

### 3.4 Bacterial surface display

Bacterial display systems present Abs fragments on the surface of live bacteria — commonly *E. coli* or *Staphylococcus carnosus* — by genetically fusing the binding domain to the outer-membrane or cell-wall anchoring proteins, thereby preserving a genotype-phenotype linkage (59, 73). This enables direct interrogation of binding properties via FACS or magnetic-activated cell sorting (MACS), while allowing for the rapid recovery of the encoding plasmids for sequencing and further engineering (74).

In *E. coli*, the high-efficiency display of single-domain Abs, such as VHHs, typically utilizes autotransporters (e.g., EhaA) or inverse autotransporters (e.g., intimin), which translocate the folded domain from the oxidizing periplasm to the bacterial surface (75, 76). Gram-positive systems, such as *S. carnosus*, employ sortase-mediated anchoring to the cell wall, allowing for robust display compatible with stringent sorting conditions (77). These formats have been extensively validated for camelid VHHs, achieving sub-nM affinities and even recovering binders missed by phage display (75, 77).

However, their small size, compact IgNAR-derived scaffold, and tolerance to oxidative folding make them strong candidates for this platform (23, 49). Multiple studies have demonstrated efficient cytoplasmic and periplasmic localization for VNARs expression in *E. coli*, engineered strains, whose design facilitates correct disulfide bond formation (78, 79).

The low cost, scalability, and compatibility with high-throughput mutagenesis, bacterial display could complement established VNAR selection platforms (phage, yeast, ribosome). Lessons from VHH campaigns suggest it could be particularly useful for quantitative affinity maturation, epitope-specific enrichment, and direct selection against cell-surface antigens (75, 77, 80).

**Table 1** compares the main characteristics of phage, ribosome, yeast, and bacterial display platforms used for VNAR library selection, highlighting differences in library size, processing speed, cost, scalability, and achievable affinity.

**Table 1:** Comparative characteristics of display platforms applied to VNAR library selection.

Display platform	Library size	Process speed	Relative cost	Display per cell/particle	Scalability	Direct affinity measurement	Achievable affinity	Affinity maturation	Application to VNAR	References
Phage display	10^9^–10¹^0^ clones	Moderate: 1–2 weeks (3–5 panning rounds)	Low	Low–moderate: few copies per phage	High: simple and inexpensive bacterial culture	No; requires ELISA or SPR post-selection	High: nM–sub-nM, pM after maturation	Yes: mutagenesis and re-panning	Yes, proven and successful	(169–171)
Ribosome display	10¹²–10¹^4^ sequences	Very fast: 3–5 days for multiple *in vitro* rounds	Moderate–high	No free display (protein bound to ribosome)	Maximum: no need for cellular transformation	No; selection based on binary binding	High: nM in practice, potential pM from diversity	Yes: epPCR between rounds	Yes, although infrequent	(41, 59, 172, 173)
Yeast display	10^6^–10^8^ transformant clones	Fast: 2–3 days of FACS (once the library is ready)	Moderate	Moderate: 10^4^–10^5^ copies of Aga2p, 1 VHH/VNAR per cell	Moderate: slower growth than *E. coli*	Yes; via FACS and antigen titration	Very high: sub-nM, pM after mutagenesis	Yes: FACS with mutated variants or selective pressure	Yes, with immune-specific libraries	(63–65)
Bacterial display	10^7^–10^9^ transformant clones	Very fast: 1–2 days for FACS or MACS	Very low	Moderate: 10²–10³ antibodies per *E. coli* cell	Very high: rapid growth of *E. coli*	Yes; via FACS	Very high: sub-nM, pM after optimization	Yes: FACS with mutated variants or selective pressure	No; unexplored potential	(174–177)

The library size for phage, yeast, and bacterial display refers to the approximate number of independent transforming clones (cells or phage particles) in the library. In contrast, the library size for ribosome display refers to the number of unique DNA/mRNA sequences present in the cell-free translation mixture and is therefore not constrained by transformation efficiency.

In summary, while phage display remains the dominant and most widely validated platform for VNAR discovery — discussed in detail in the previous section — alternative display systems have emerged that offer complementary capabilities for specific selection needs, such as: Ribosome display allows for fully *in vitro* selection from ultra-large libraries without transformation bottlenecks (59); yeast surface display combines eukaryotic folding with quantitative affinity measurements and multiparametric sorting (63); and bacterial display, although not yet applied in published VNAR selection campaigns, presents a technically compatible, low-cost and rapid option, extensively demonstrated in related single-domain Abs formats (75). Together, these platforms expand the methodological landscape for VNAR engineering, offering opportunities to tailor selection strategies based on target properties, required throughput, and downstream functional assays.

## 4 Generating VNAR sources and libraries

There are various methods for identifying and isolating target-specific sdAbs. VNARs can be amplified from cDNA (sdAbs are encoded from individual white blood cell genes), expressed in prokaryotic or eukaryotic systems, and generated from immunized, non-immunized (naïve) sharks, or from synthetic or semi-synthetic libraries (81–84).

**Figure 4:**
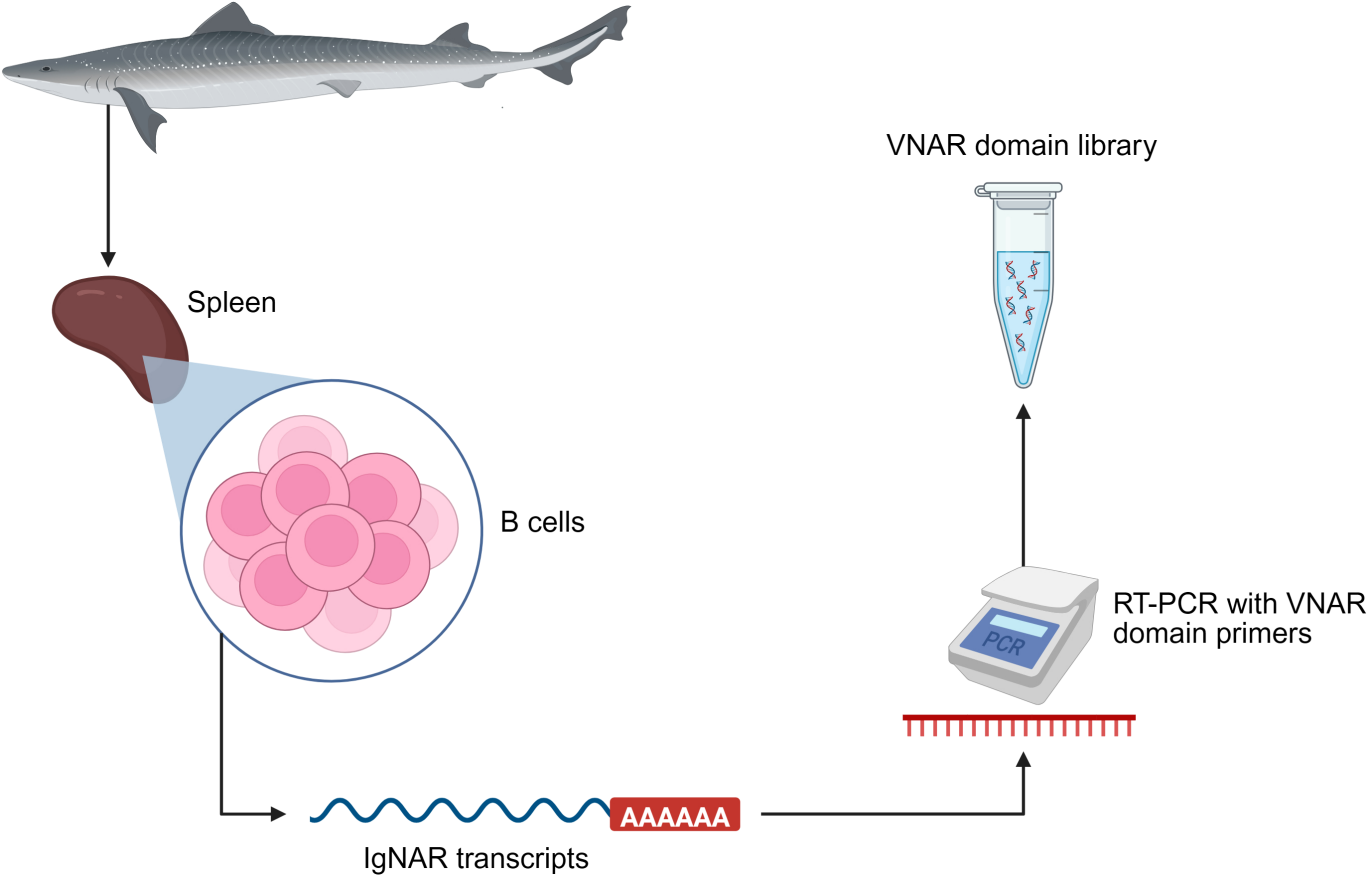
Schematic representation of the generation of a VNAR domain library. Total RNA is extracted from the spleen, which is the main secondary lymphoid organ where antibody-producing B cells are activated and mature. Subsequently, using reverse transcription PCR (RT-PCR) and specific or degenerate primers, the VNAR domains of the IgNAR transcripts contained in the total RNA are amplified. The PCR products result in a DNA library of VNAR domains which allows for downstream applications such as generation of synthetic and/or semi-synthetic libraries or the use of *in vitro* technologies. Created with BioRender.com.

The functional and structural versatility of VNARs depends on the availability of diverse and functional repertoires of these molecules. In modern biotechnology, these repertoires are obtained from natural sources (**Figure 4**) (immunized or naïve animals) or synthetic methods, thus generating genetic libraries that can be explored using selection platforms such as phage display, yeast display, or other *in vitro* technologies (60, 85). These libraries are essential for identifying VNARs with desired characteristics, such as high affinity, stability, or low immunogenicity. The primary sources and strategies for their generation are described below:

### 4.1 VNARs derived from immunized sharks

Immune VNAR libraries are generated by immunizing sharks with a specific antigen, eliciting an *in vivo* affinity-matured IgNAR repertoire enriched for high-affinity binders. The most widely used model is the nurse shark (*Ginglymostoma cirratum*), and other species have also yielded successful immune libraries, including the white-spotted bamboo shark (*Chiloscyllium plagiosum*), the ornate wobbegong (*Orectolobus ornatus*), horn shark (*Heterodontus francisci*), and the spiny dogfish (*Squalus acanthias*). The selection of host species is crucial; for example, bamboo sharks exhibit, particularly robust IgNAR recall responses strong IgNAR recall responses (86). In contrast, the small-spotted catshark (*Scyliorhinus canicula*) often displays an uncoupling of IgNAR and IgM responses potentially limiting immune library quality (87).

Although immunization protocols vary in dose, route, adjuvant, and boost frequency, the most common approach uses an initial injection (often with Freund’s complete adjuvant) followed by biweekly or monthly boosts over 2–6 months (36, 78, 82). Some regimens demonstrate species-specific optimization, for instance, biweekly subcutaneous boosts in bamboo shark have produced higher VNAR titers. Following the final boost, lymphoid tissues such as spleen, thymus, and epigonal organ, or peripheral blood, are collected for mRNA isolation. B-cell mRNA is reverse-transcribed to cDNA, and IgNAR variable domains are amplified using degenerate or framework-specific primers, then typically cloned into filamentous phage display systems — by far the most widely used platform for immune VNAR libraries — to generate libraries normally ranging from 10^7^ to 10^8^ independent clones (19, 78). However, alternative display formats such as yeast and ribosome display have also been employed in certain studies (85, 88).

The resulting repertoires are heavily biased toward the immunogen but benefit from *in vivo* affinity maturation. Somatic hypermutation — primarily in CDR3 and occasionally in CDR1 or HV2 — drives the selection of high-affinity, structurally stable variants (36, 89). This process often produces long or cysteine-rich CDR3 loops, stabilized by interloop disulfide bonds that enable recognition of recessed epitopes inaccessible to conventional Abs (89, 90). As a result, immune VNARs are generally well-folded, soluble, and thermally stable, with affinities often in the nM or sub-nM range without further *in vitro* maturation.

Immune VNAR libraries have demonstrated remarkable versatility, generating high-affinity binders (typically in the nM to sub-nM) against an extensive spectrum of targets, including soluble proteins, cell-surface ligands, pathogen antigens, and cytokines. Reported applications span domains specific for ICOSL (83), necrosis tumoral factor (TNF) alpha (α) (82), *Plasmodium falciparum* biomarkers (91), viral glycoproteins (92), and bacterial virulence factors (93). This broad target coverage highlights the unique potential of VNARs as compact, stable, and robust binding domains suitable for therapeutic intervention, diagnostic platforms, and fundamental research.

The principal advantage of immune VNAR libraries is their inherently high functional quality: binders are naturally pre-selected *in vivo* for affinity, specificity, correct folding, and stability. Yet, these strengths come with essential constraints. Because immunizing antigens shape the repertoire, immune libraries often exhibit a narrow target bias and show limited capacity to generate binders against unrelated molecules. A well-documented example is the nurse shark anti-lysozyme library, in which immunization with hen egg-white lysozyme yielded multiple high-affinity clones against the intended target, but only a single weak binder against the unrelated protein ovalbumin. These limitations have spurred increasing interest in naïve, synthetic, and semi-synthetic VNAR libraries, which offer broader diversity and greater flexibility for discovery against novel antigens (4, 43).

In addition, the generation of immune libraries is resource-intensive, as immunization protocols may extend over several months, necessitate repeated handling of live animals under strict ethical and regulatory oversight, and require specialized facilities, particularly when working with large species such as nurse sharks (86). Interspecies variation in IgNAR responses further means that not all sharks are equally suitable donors, as exemplified by the case of the small-spotted catshark. Efforts to address these constraints include using smaller, more manageable species such as bamboo sharks, which mature quickly, are inexpensive to maintain, and have yielded repertoires comparable in both diversity and affinity to those from larger species. By refining immunization protocols and leveraging species biology, immune VNAR libraries remain a gold-standard platform for producing high-performance binders to defined antigens, with a proven track record in both basic research and translational applications.

### 4.2 Naive (non-immunized) libraries

Naïve VNAR libraries represent the unselected antigen-binding repertoire of sharks before deliberate immunization. Their diversity originates from V(D)J recombination and junctional diversification, with most sequence heterogeneity concentrated in the often elongated, cysteine-rich CDR3 loop. In contrast, CDR1 and the hypervariable HV2/HV4 loops remain germline-encoded mainly in the absence of antigenic stimulation (25, 94). Unlike immune libraries, which are biased toward a single immunogen, naïve collections retain a broad representation of the natural repertoire and can be repeatedly screened against unrelated antigens. This is particularly advantageous when immunization is infeasible or unsafe (e.g., poorly immunogenic or toxic antigens) or when rapid binder generation is required (60, 95).

Construction typically involves pooling lymphoid tissues or peripheral blood from multiple non-immunized donors to maximize germline representation. In large-scale efforts, several nurse sharks have been used, while data from the bamboo shark and small-spotted catshark species inform donor choice and baseline isotype diversity (25, 86, 87). VNAR coding sequences are amplified from B-cell mRNA with primers targeting conserved framework motifs to recover all subtypes (I–IV), including cysteine-enriched variants (60). High-capacity cloning into phage display vectors — often via PCR-extension assembly and self-ligation (EASeL) — reduces cloning bias and supports library complexities of up to 10^9^–10¹^0^ independent clones (25). Quality control by Sanger sampling and deep sequencing assesses loop-length distributions, cysteine patterns, and germline usage, with large naïve nurse shark libraries reporting >80% unique sequences and balanced subtype representation. Comparable methodologies in white-spotted bamboo shark confirm scalability and efficient downstream screening (96).

Functionally, naïve VNAR libraries have delivered binders to diverse targets. Specifically, from a naïve nurse shark-derived library comprising approximately 1.2 ×10¹^0^ unique clones, VNARs that bind to the SARS-CoV-2 S2 subunit were isolated, including neutralizers with broad cross-reactivity against divergent β-coronaviruses (97). Earlier, a smaller (~10^7^) dogfish library provided cholera-toxin-specific VNARs that retained activity after extended exposure to 95°C, demonstrating exceptional scaffold stability (98). With increasing donor breadth and library size, selections from naïve nurse-shark repertoires have also produced binders to mammalian cell-surface targets relevant to oncology and immunomodulation, extending applications beyond infectious disease (25). These and other selection campaigns illustrate the functional versatility of naïve VNAR repertoires, which is underpinned by their intrinsic numerical and structural diversity. The breadth of naïve repertoires reflects both numerical and structural diversity. Long CDR3 loops — often stabilized by non-canonical disulfides — permit access to recessed or conserved epitopes. At the same time, the phylogenetic distance between sharks and mammals expands the accessible antigenic space toward conserved mammalian proteins (25, 99).

In the absence of antigen-driven affinity maturation, initial hits from naïve libraries typically exhibit affinities ranging from micromolar (µM) to high nM, with limited diversity outside CDR3. Targeted *in vitro* maturation — through CDR1, HV2/HV4, or CDR3 mutagenesis — can elevate affinities to immune-derived levels (95). Although some epitopes may be absent or under-represented, the combined use of multiple donors, large library sizes, and informed species selection effectively mitigates this limitation in practice (96).

### 4.3 Semi-synthetic libraries

Semi-synthetic VNAR libraries represent a hybrid strategy that integrates the inherent structural robustness of natural shark VNAR frameworks with *in vitro*-engineered diversity in antigen-binding loops. Unlike immune libraries, they bypass the need for animal immunization, enabling rapid selection against virtually any target, including poorly immunogenic, highly conserved, or toxic antigens (100, 101). Typically, they start from one or more well-expressed and stable VNAR scaffolds — often Type II or engineered Type II – like frameworks — into which synthetic diversity is introduced, most commonly in CDR3 and, in some cases, CDR1 or hypervariable loops HV2/HV4 to modulate paratope topology (67, 71). Type II VNARs commonly feature a CDR1–CDR3 disulfide; semi-synthetic designs may remove or restrict noncanonical cysteines to simplify folding and expression. The design phase enables precise control over CDR3 length, amino acid composition, and cysteine content, allowing either the prevention of unwanted disulfide bonds or the deliberate introduction of stabilizing loops (19).

Library construction is generally achieved using site-directed or saturation mutagenesis with degenerate oligonucleotides, or by trinucleotide-directed synthesis to avoid stop codons and codon bias (102). Reported libraries have achieved effective diversities on the order of 10^8^–10^9^ functional clones, comparable to high-quality naïve VNAR repertoires but with a high fraction of correctly folded, expressible variants (41, 103). Diversity is often organized into sub-libraries based on CDR3 length or the presence of specific motifs, thereby maximizing coverage of conformational space coverage (71). For screening, phage display remains the most widely adopted platform due to its scalability and versatility offering enormous diversity (50, 71).

However, yeast surface display has gained traction by enabling quantitative selection via flow cytometry and controlling surface expression through dual-display systems (72). Integrating next-generation sequencing (NGS) into selection workflows enables the detailed tracking of clonal enrichment and the prioritization of promising variants eliminating the need for extensive low-throughput screening (71, 72). Semi-synthetic VNAR libraries have yielded functional binders against a broad range of targets. Notable examples include anti-idiotypic VNARs specific for therapeutic Abs such as cetuximab and matuzumab, selected from bamboo shark Type II scaffolds and achieving sub-nM affinity in Fc fusion formats (71). VNARs have also demonstrated functional versatility beyond classical antigen recognition: for instance, domains specific for B-cell activating factor (BAFF) can inhibit B cell development through molecular mimicry, highlighting their potential as modulators of immune signaling pathways (104). In parallel, the targeted diversification of hypervariable loop 2 HV2 on a fixed CDR3−specific VNAR scaffold has enabled bispecific single−domain formats capable of engaging two distinct epitopes simultaneously, thereby expanding the structural and functional adaptability of VNAR-based therapeutics (24).

### 4.4 Synthetic libraries

Fully synthetic VNAR libraries push the concept of *in vitro* diversity to the extreme by designing VNAR sequences from scratch rather than relying on existing antigen receptor sequences. In practice, synthetic VNAR libraries often begin with a limited set of engineered consensus scaffolds, such as a streamlined Type II VNAR framework optimized for stability and devoid of structural liabilities (105). Onto this minimal template, extensive genetic diversity is introduced at the genetic level. In early synthetic VNAR libraries, diversification was applied almost exclusively to the CDR3 loop, using degenerate codons to generate large combinatorial diversity without reference to any single shark repertoire (106). More recent synthetic designs have expanded this strategy to include variation in CDR1 in addition to CDR3, in some cases introducing non-canonical cysteines in one or both loops to explore a broader range of disulfide patterns and paratope geometries. These libraries, often built on Type II or IIb frameworks, maintain a large size — up to ~2x10¹^0^ independent clones — while applying targeted constraints to preserve structural integrity (105).

The aim is to cover a broad combinatorial space of paratope configurations, potentially exploring sequence variants that have never appeared in nature. This scale of diversity greatly surpasses what can realistically be obtained from the immune repertoire of a single animal, allowing for a much more comprehensive exploration of sequence space.

However, managing functional quality in a purely synthetic library is challenging. Even so, over 90% functional clones have been reported from successful synthetic Abs libraries, which have required careful design and validation processes (107).

Unlike semi-synthetic libraries, which rely on pre-validated frameworks, a purely synthetic approach carries the risk of generating a substantial fraction of non-functional clones, such as misfolded or aggregated molecules, due to incompatible mutations introduced during randomization. To mitigate this, designs often impose constraints to maintain expressiveness and proper folding, including the use of NNK instead of NNN codons to minimize premature stops and the employment of amber-suppressor strains during display (108), or the incorporation of non-amber stop codons into the template that are removed only after successful mutagenesis to prevent display of the unmodified scaffold. Additional strategies include building multiple sub-libraries to vary CDR3 length and cysteine content, thereby covering different disulfide patterns and paratope geometries. With careful design and quality control, synthetic VNAR libraries can achieve high proportions of correctly folded, display-competent clones — for example, approximately 86% of the clones in one synthetic VNAR library (34).

Since synthetic VNAR libraries lack immune selection, initial hits typically exhibit moderate binding affinities, with K_d_ values often ranging from low nM to several hundred nM. As observed in analogous synthetic single-domain libraries (109), direct retrieval of sub-nM binders is uncommon but possible, and these rare clones typically do not require further optimization. To improve affinity, researchers frequently apply *in vitro* maturation methods such as epPCR or targeted CDR mutagenesis or use multivalent display formats to enhance apparent binding. Despite this, large and well-designed synthetic libraries can yield potent VNAR binders, sometimes matching the performance of affinity-matured Abs (110).

Synthetic VNAR libraries offer a compelling combination of rapid deployability and programmability — facilitating selection against virtually any antigen without reliance on animal immunization or natural repertoires. The ability to design diversity *in silico* enables the exclusion of problematic motifs (e.g., glycosylation sites, protease-sensitive residues, oxidation-prone cysteines). It permits the incorporation of non-natural amino acids to tailor paratope characteristics beyond natural constraints (41, 111–113).

However, the lack of natural selection mechanisms poses significant challenges. Synthetic libraries may yield a substantial fraction of non-functional, misfolded, or poorly expressed clones due to randomization biases (114). Moreover, fully synthetic repertoires cannot replicate the co-evolved structural features inherent to natural VNARs, which contribute to stability and specificity (115). Achieving high-affinity binders typically demands very large library sizes to ensure sufficient coverage of sequence space (116). In addition, even selected clones may carry hidden liabilities — such as reduced biostability or polyreactivity — necessitating further rounds of maturation before *in vivo* or diagnostic application. **Table 2** summarizes the main characteristics of immune, naïve, semi-synthetic, and synthetic VNAR libraries, highlighting their advantages, limitations, and recommended selection platforms.

**Table 2:** Key features of immune, naïve, semi-synthetic, and synthetic VNAR libraries.

Type	Origin	Typical size	Expected initial affinity (K_d_)	Time to hits	Advantages	Limitations	When to choose	Recommended selection platforms	References
*Immune*	B lymphocytes from sharks immunized with the antigen	10^7^–10^9^	Typically, nM; sometimes sub-nM without extensive *in vitro* maturation	Several weeks–months	High hit rate: clones are already specific, stable, and well-folded	Requires animals and time; biased toward immunizing antigen; unsuitable for toxic or poorly immunogenic antigens	Therapeutic programs where immunization is possible and starting with high affinity is desired	Phage, Yeast (FACS), Ribosome	(21, 60)
*Naïve*	B lymphocytes and repertoire without immunization or modification	10^8^–10¹^0^	Typical µM or nM; improves with *in vitro* maturation	1–3 weeks	Not biased to a single antigen; useful for difficult or toxic targets	Higher proportion of non-functional clones; requires affinity maturation	Rapid multi-antigen exploration or when immunization is not possible	Phage, Yeast (FACS for off rate), Ribosome	(21, 25, 60, 98)
*Semi-synthetic*	Stable natural FRs + artificially diversified CDRs	10^8^–10¹^0^	10–300 nM after 2–3 rounds; solid base for maturation	1–3 weeks	Good balance between scaffold stability and useful diversity; does not require animals	Excessive variation may produce misfolded sequences	When functional quality is needed without immunization and with targeted diversity in CDR3	Phage, Yeast (FACS), Ribosome	(23, 36, 71)
*Synthetic*	*De novo*–designed FRs + fully synthetic diversity	10^9^–10¹²	Often 50–500 nM initially; can reach nM–sub-nM with maturation	1–3 weeks	Large and controlled coverage; no animals; adaptable to any target	Requires validation and functional filtering to remove misfolded variants	High-throughput screening or when covering large CDR3 space with clear design rules is desired	Ribosome (XL), Phage (XL), Yeast (FACS for kinetics)	(34, 60, 106)

## 5 Structural characteristics required in biomedicine

VNARs contain only two canonical CDRs but feature two extra highly variable loops. This unique architecture, comprising a small, simple domain and four diverse loops, contributes to its distinctive structures and antigen-binding mechanisms (60). Shark VNARs may have unique therapeutic potential, offering advantages that make VNARs a superior therapeutic platform. Briefly, the VNARs exhibit several structural advantages that enable their application in biomedicine.

### 5.1 Reduced size and associated functional advantages

VNARS have smaller molecular sizes, allowing for efficient tissue diffusion and cellular uptake. VNARs feature a distinctive “finger-like” paratope architecture characterized by extended and prominent CDR3 loops. This unique three-dimensional structure enables them to penetrate and bind to hidden epitopes within deep catalytic leaves and hidden binding pockets that are often inaccessible to larger, conventional Abs, providing improved access to target sites and allowing superior extravasation from blood vessels, for a deeper and homogeneous penetration into dense and poorly vascularized tissues (28, 49). This characteristic is particularly advantageous in oncological applications, where VNARs can effectively target antigens residing within the tumor microenvironment that are largely inaccessible to bulky conventional monoclonal Abs.

### 5.2 Exceptional structural stability

VNARs exhibit remarkable thermostability, maintaining functional integrity even at 80°C or 95°C (117). This exceptional robustness makes them suitable for diagnostic and therapeutic applications in challenging environments, including tropical regions where temperature stability is crucial for drug efficacy and storage.

### 5.3 High solubility

VNARs display high solubility in aqueous solutions, facilitating their formulation and handling compared to conventional Abs. This property simplifies pharmaceutical development processes and potentially reduces manufacturing complexities associated with protein aggregation and precipitation (17). Experimental studies have shown that VNAR domains remain functional and largely monomeric after exposure to elevated temperatures, repeated lyophilization/rehydration, and long-term storage in liquid formats, under conditions where conventional IgG frequently loses activity or tends to aggregate (118). In line with this, anti-TNF-α VNAR formats have been shown to retain their bioactivity and suitable biophysical behavior after formulation and storage, supporting the notion that VNAR scaffolds possess favorable solubility and stability profiles for therapeutic development (119). Regarding the development capabilities of VNARs, a diversity of formulations can be achieved. Specifically, designs can be generated with higher protein concentrations, alternative routes of administration, and the incorporation of VNARs in multivalent, multispecific, or fusion formats. This can minimize the risk of liabilities related to solubility during manufacturing and long-term storage of VNARs.

### 5.4 High versatility and genetic modifiability

VNARs can be efficiently produced in microbial organisms such as bacteria (*E. coli*) and yeast (120). This compatibility with prokaryotic expression systems makes VNARs cost-effective tools for research, industrial applications, and therapeutic development (78). Additionally, recombinant VNAR offers superior reproducibility and batch-to-batch consistency with significant yields. This predictable production profile reduces manufacturing risks and ensures consistent therapeutic product quality, addressing critical concerns in biopharmaceutical development (63).

Also, VNARs can be fused with other proteins or peptides to create diverse therapeutic formats, including chromobodies for high-resolution bioimaging, bispecific and multispecific constructs, antibody-drug conjugates, and chimeric antigen receptor T-cell (CAR-T) therapies (121). This exceptional modifiability dramatically expands their therapeutic application spectrum, enabling the development of sophisticated, multi-functional therapeutic agents.

## 6 Uses of VNARs in biomedicine

### 6.1 Use of the VNARs in immunodiagnostics

The advantages described above for VNARs support their application in immunodiagnostics and biomarkers. These diagnostic tests, especially those used in remote or resource-limited areas, can be affected by exposure to elevated temperatures and humidity. Consequently, VNARs are increasingly utilized in diagnostics, pharmaceutical development, and biomedical research. In addition, studies have shown that VNARs can bind antigens to form high-affinity complexes, allowing them to recognize and bind target antigens efficiently, contributing to diagnostic clinics (49, 117).

Comparative studies have conclusively shown that while conventional mAbs immobilized on nitrocellulose strips (the basis of most Rapid Diagnostic Tests) rapidly lose activity at elevated temperatures (122), VNARs retain nearly 100% of their binding function even after weeks of storage at 45°C (118). This exceptional thermostability revolutionizes point-of-care testing by eliminating the need for a refrigerated cold chain, a significant logistical and economic barrier in global health.

On the other hand, studies have demonstrated this capability using an apical membrane antigen 1 (AMA1)-specific IgNAR Abs against *P. falciparum*, revealing that extended CDR3 loops penetrate deep hydrophobic clefts and contact conserved residues across parasite species, providing insights for targeting otherwise inaccessible pathogen epitopes (123, 124).

VNARs have also been demonstrated to target biomarkers derived from viral diseases such as Ebola hemorrhagic fever (EHV), which is the cause of death by the Ebola virus (125, 126). Two VNARs (DSTL096 and DSTL097) have been successfully isolated from immunized phage display libraries specific to the Ebola virus nucleoprotein (92, 127).

VNARs have been used as biomarkers for toxins such as cholera toxin, botulinum toxin A (BoNT/A), ricin, and staphylococcal enterotoxin B (SEB) (50, 98), improving the speed and accuracy of cholera toxin (CT) detection in many tropical and subtropical developing countries.

In the case of the hepatitis B virus (HBV), it is a hepatotropic DNA virus capable of establishing persistent chronic infections, affecting approximately 3.9% of the global population, representing a significant public health burden despite the availability of effective vaccines for over four decades. Its clinical spectrum ranges from acute hepatitis to severe chronic outcomes such as cirrhosis, liver failure, and hepatocellular carcinoma, underscoring the need for improved diagnostic and therapeutic strategies (128).

Researchers have developed a rapid, highly sensitive diagnostic assay based on VNARs that can detect HBV antigens (HBsAg) in serum samples with high accuracy. Studies have generated VNARs from the white-spotted bamboo shark (*Chiloscyllium plagiosum*) immunized with HBsAg, leading to the construction of a phage display library. Three candidates expressed in *E. coli* demonstrated strong specificity and binding capacity, and pairwise evaluation in sandwich ELISA assays confirmed their diagnostic potential, laying the groundwork for the development of novel, efficient VNAR-based detection platforms for HBV (128).

This assay has the potential to improve the diagnosis of HBV infection, particularly in resource-limited settings where traditional diagnostic methods may not be readily available. VNARs have also been used to detect CT with high accuracy. Indeed, the anti-CT VNARs was able to detect concentrations as low as 1ng/mL of CT in a Luminex-based sandwich assay (98).

The use of shark-derived VNARs as a diagnostic tool for viral infections is a promising area of research with numerous applications. The development of rapid, highly sensitive diagnostic assays based on VNARs has the potential to improve the speed and accuracy of virus diagnosis, particularly in resource-limited settings where traditional diagnostic methods may not be readily available (98).

### 6.2 Immunotherapy and cancer

Solid tumors consist of tumor cells along with vasculature, extracellular matrix, stromal cells, and immune cells. The tumor microenvironment comprises a substantial portion of the total tumor mass. This microenvironment is characteristically dense and solid, with abundant components that include epitopes that may be partially obscured by factors such as glycosylation, internalization, or increased extracellular matrix expression. High levels of dense and rigid extracellular matrix can serve as a barrier, potentially limiting access of therapeutic agents to cells and contributing to tumor chemoresistance (129).

VNARs are considered potential therapeutic agents for cancer treatment in this setting. They may inhibit tumor cell proliferation, interfere with signaling via growth factor receptors (GFRs), induce apoptosis, or target tumor cells for immune system recognition and elimination. The small size and extended, flexible CDR3 loops of VNARs facilitate access to regions that traditional Abs may not reach, which can be advantageous for targeting antigens in poorly vascularized tissues or other hard-to-reach areas.

Studies have demonstrated that VNARs can interfere with angiogenesis (the formation of new blood vessels that sustain tumors). Besides, they have been designed for different pathologies, such as breast and gastric cancer, lung and colorectal cancer and tumor angiogenesis blockade by interfering with the vascular endothelial growth factor (VEGF) pathways (51).

CARs are synthetic receptors consisting of an extracellular domain, a hinge region, a transmembrane domain, and intracellular signaling domains (such as CD3-zeta, CD28, and 41BB) that initiate T-cell activation. These CARs enable MHC-unrestricted recognition of cell surface components, directly bind tumor antigens, and trigger an antitumor T-cell response (130–135). Notably, CAR-T cells targeting the CD19 antigen have demonstrated clinical efficacy in patients with advanced B-cell lymphoma and have received approval by the U.S. Food and Drug Administration (FDA) (136, 137).

Despite success in hematologic cancers, translating CAR-T therapy to solid tumors is more difficult, mainly due to:

- Lack of appropriate antigenic targets: Solid tumor antigens are not always unique and accessible, as the tumor microenvironment is denser and the proteins on the surface are often glycosylated, which hinders the penetration of drugs.
- Immunosuppressive tumor microenvironment (TME): The TME of solid tumors is complex and immunosuppressive, hampering CAR-T persistence and infiltration. Conventional CAR-T constructs, which use single string variable fragments (scFv) for antigen recognition, have shown limited success in solid tumors, partly because of poor persistence and infiltration into this dense TME.

SdAbs provide key advantages for CAR-T therapy in solid tumors due to their small size and high stability, which enhances CAR expression on T-cell surfaces. Their compact structure facilitates better penetration into dense tumor microenvironments, allowing access to tumor cells beyond the extracellular matrix. Additionally, their unique CDR3 loop enables binding to occluded epitopes inaccessible to conventional Abs, making them effective against antigens masked by glycosylation or dense extracellular structures (138–140).

VNARs have been integrated into CAR-T cell strategies targeting cancer. Notably, shark VNARs possess unique characteristics that distinguish them from camel VHHs, including greater diversity.

They are evolutionarily derived from an ancient single domain that functions as a variable domain in B-cell and T-cell receptors (60, 141). Studies on programmed death-ligand 1 (PD-L1 or CD274) have demonstrated that it is often overexpressed across various tumor types due to oncogenic signaling, and its expression is further increased by pro-inflammatory factors such as interferon-gamma (IFN-γ) within the immunoreactive tumor microenvironment. Research has shown that PD-L1 on tumor cells can promote T cell tolerance and aid immune evasion by interacting with PD-1 on T cells, which might be a key factor behind the limited effectiveness of CAR-T cells against solid tumors. Clinically, antibody-based inhibitors of the PD-1/PD-L1 axis have demonstrated sustained antitumor activity, especially in melanoma, non-small cell lung cancer, and renal cancer. Studies have expanded the diversity of the shark VNAR repertoire by constructing a semi-synthetic VNAR phage library characterized by a randomized CDR3 region of 18 amino acids in length. Of the three cross-reactive binders obtained, only VNAR B2 was able to block the interaction between human PD-L1 and PD-1 functionally. Notably, B2-based CAR-T cells effectively inhibited tumor growth in murine xenograft models of triple-negative breast cancer (TNBC) and hepatocellular carcinoma (HCC). Furthermore, the combination of CAR-T cells with anti-PD-L1 CAR (B2) and anti-GPC3 CAR demonstrated superior efficacy compared to single-antigen-targeted CAR-T cells in the murine HCC model, highlighting the therapeutic potential and feasibility of shark VNAR-based CAR-T cells targeting PD-L1 in solid tumors (140).

The VNAR-based CAR-T cell therapy represents a promising approach for treating breast and liver cancers, establishing a foundation for the potential application of PD-L1-targeted CAR-T cells either as monotherapy or in combination with tumor-specific therapeutic strategies in clinical settings. Ongoing research in this field underscores its dynamic development, and additional clinical trials are anticipated to substantiate further and validate its therapeutic potential in oncology.

However, significant challenges remain, particularly the need to humanize the VNAR sequence and optimize its relatively short *in vivo* half-life. Despite these hurdles, VNARs offer a substantial economic advantage, with production costs far lower than those of conventional Abs drugs, which support their potential for broader clinical translation.

### 6.3 Infectious diseases

The application of VNARs in infectiology, especially in virology, has gained considerable momentum, particularly against viruses that harbor highly conserved or difficult-to-access sequences. The ability of VNARs to target highly conserved cryptic epitopes makes them powerful tools against rapidly mutating pathogens.

Notable examples of VNARs targeting viruses include: severe acute respiratory syndrome coronavirus 2 (SARS-CoV-2), where VNARs target the receptor-binding domain (RBD) of the spike protein (142); HBV, specifically targeting the hepatitis B surface antigen (HBsAg) (128); and influenza A virus, recognizing the matrix protein 2 (M2) ion channel (143).

The M2 protein of influenza A virus is an integral membrane protein composed of an ectodomain, which in turn consists of a transmembrane domain and an intracellular helix. Furthermore, in its active physiological state, it assembles into a tetramer functioning as a proton channel essential for viral replication, making it a key target protein for antiviral development. Recent studies highlight that effective Ab generation requires antigens in their native conformation. Thus, the tetrameric form of M2, maintained through nanodisc assembly, has been used for nanobody screening using phage display libraries. This approach led to the identification of the VNAR AM2H10, which showed strong affinity for the native M2 tetramer and demonstrated inhibitory activity against both wild-type M2 channels and amantadine-resistant strains. These results underscore the therapeutic promise of using nanodisc-based systems to develop functional nanobodies targeting membrane proteins and support ongoing preclinical efforts toward M2-specific nanobody vaccines. Although research on VNARs remains limited to a few shark species, the findings establish a solid foundation for their potential as innovative drug candidates against influenza (143).

The University of Wisconsin-Madison, through Aaron LeBeau’s laboratory, constitutes a critical academic collaborator applying VNAR technology to infectious diseases and cancer. Researchers have tested shark VNARs against SARS-CoV-2 and identified three effective candidates, including one named 3B4, which shows promise. Specifically, the 3B4 VNAR binds to a highly conserved groove on the viral spike protein, enabling it to neutralize not only SARS-CoV-2, but also SARS-CoV-1 and the distantly related Middle East Respiratory Syndrome coronavirus (MERS-CoV). This binding site remains unchanged across SARS-CoV-2 variants, including Delta, and initial models suggest 3B4 would remain effective against Omicron, making it a promising broad-spectrum antiviral candidate (105).

### 6.4 Autoimmune and inflammatory disorders

#### 6.4.1 Rheumatoid arthritis

VNARs have been developed against key inflammatory mediators, such as TNF-α, which have shown superior efficacy compared to adalimumab in preclinical studies (82). VNARs targeting Inducible T-Cell Costimulator Ligand (ICOSL) have demonstrated the ability to inhibit T-cell proliferation and reduce inflammation in animal models of arthritis.

The use of the horn shark anti-TNF-α VNAR has been reported. This platform offers a unique competitive advantage over mammalian-derived therapies by enabling the recognition of epitopes that are immunologically inaccessible to the immune system of mammals. This provides an opportunity to develop novel, high-affinity Abs with therapeutic mechanisms unattainable through conventional approaches, potentially addressing unmet clinical needs in patients who do not respond to current anti-TNF therapies (82).

#### 6.4.2 Blood-brain barrier

The blood-brain barrier (BBB) is a physiological barrier that can prevent both small, complex drugs from reaching the brain to exert a pharmacological effect, posing a significant obstacle for the development of biopharmaceuticals with therapeutic effects within the central nervous system. However, for the treatment of neurological diseases, drug concentrations at the target site are a fundamental parameter for therapeutic effect. Novel strategies have been developed to circumvent the BBB, including receptor-mediated transcytosis via the transferrin receptor 1 (TfR1), utilized by VNAR shuttles such as TXB2 (37). At this point, advancements in drug delivery across the BBB will require appropriately designed and powered clinical studies (144).

Passive immunotherapy has been increasingly used over the last decade in several diseases such as cancer and inflammation. Diffusion of conventional Abs is restricted in tissues due to their large size and by the blood-tumor barrier (BTB) and BBB, limiting access to the tumor center (145–147). Several studies have explored the different options of sdAbs, such as VHHs and VNARs, which have already been taken to reach the brain, allowing them to be used as therapeutic, diagnostic, or transporter tools (148, 149).

An example has been the establishment of a functional selection method to identify high-affinity single-domain antibodies to the transferrin receptor 1 (TfR1) with efficient biotherapeutic delivery across the BBB. Receptor-mediated transport systems, such as TfR1, have been exploited to deliver a wide range of biological products to the brain in a non-invasive manner (37). TfR1 is the most extensively characterized receptor for the uptake of iron-loaded transferrin and the subsequent transfer of iron to the brain. Despite clear advances, several features of monoclonal anti-TfR1 Abs used as BBB carriers have disadvantaged their clinical development, because monoclonal Abs can cause anemia by target-mediated lysis of TfR1-rich cells (150). For resolving these problems, the researchers developed a synthetic phage display library based on VNARs named TXB2. TXB2 is a high affinity, cross-species VNAR Abs targeting the TfR1 extracellular domain (TfR1-ECD), capable of traversing the BBB without interfering with transferrin or ferritin binding. As a high-affinity, bivalent Fc fusion protein, TXB2 quickly crosses the BBB and demonstrates a favorable pharmacokinetic and safety profile. The small size of VNARs enables their use in transport systems that could revolutionize treatment of neurodegenerative diseases, brain cancers, and other central nervous system (CNS) disorders by allowing reliable passage across the BBB for various therapeutic payloads (37).

#### 6.4.3 Corneal penetration

The smaller size, combined with their inherent stability and solubility, makes them especially useful for therapeutic purposes. These unique physical properties give VNARs clear advantages over traditional Ab types in challenging biological settings (17).

As well as drug transport across the BBB has presented a significant challenge in medicine; similarly, the cornea acts as a physical barrier to the delivery of biopharmaceuticals with therapeutic effects in the eye. VNARs have demonstrated the ability to overcome this obstacle. Several examples have been provided that explain the specialized structure of the cornea, and the administration of VNAR via topical drops have been evaluated in mouse models with corneal abrasions.

These studies have shown that VNARs can penetrate the cornea, overcoming the limitations of traditional Abs that often require intravitreal injection. This invasive procedure may carry risks, including infection and retinal detachment (51).

Autoimmune uveitis is a severe, chronic inflammatory disease and a significant cause of vision loss worldwide. This condition is characterized by a rapid and debilitating inflammation of the uvea, the pigmented and vascular structures of the eye. It’s estimated that 70% of uveitis cases are non-infectious, primarily presenting as an acute manifestation of an underlying autoimmune condition, in which T-cell activation plays a critical role in its development. Incidences vary widely, from 38–200 per 100,000 people in the Western world to 730 per 100,000 in India, and up to 35% of patients can suffer from marked visual loss (83). First-line therapy for patients with active uveitis is corticosteroids because of their rapid effect and the flexibility in the choice of their delivery — locally to the eye or systemically. However, long-term corticosteroid treatment is associated with the risk of various adverse events, including cataract, glaucoma, diabetes, cushingoid changes, hypercholesterolemia, and osteoporosis (151). When conventional treatments fail to control inflammation (in refractory patients), biological agents are an alternative. Adalimumab (Humira^®^) is a commonly used anti-TNF-α agent for posterior uveitis, having shown significant improvement in refractory patients and a reduced risk of “vision loss.” Although many patients have benefited from anti-TNF therapy, a considerable number have reported serious side effects with prolonged systemic administration (152).

Specific VNARs can recognize the human inducible T-cell costimulatory ligand (ICOSL). ICOSL plays a crucial role in T-cell activation, having been isolated from an immunized nurse shark phage library. A mouse anti-ICOSL VNAR Fc construct, tested in a murine model of interphotoreceptor retinoid-binding protein (IRBP)-induced uveitis, demonstrated high affinity for ICOSL and high corneal penetration. The results of this study were the first demonstration of the efficacy of a VNAR binding domain in a clinical disease model, revealing a marked reduction in inflammation. This highlights the potential of VNARs for treating autoinflammatory conditions (83).

Vascular endothelial growth factor A (VEGFA), specifically the VEGF165 isoform, stimulates the formation of new blood vessels (angiogenesis). Under low-oxygen conditions, its overexpression triggers pathological angiogenesis, resulting in fragile and defective vessels. This leads to hemorrhages, fluid leaks, and damage to the retina and other eye structures, which can cause vision loss. For this reason, VEGF165 is a key therapeutic target for treating eye diseases like macular degeneration and diabetic retinopathy. VNARs can penetrate the ocular surface without causing abrasion or discomfort and possess the potential to become novel drug candidates for the treatment of ocular vascular diseases.

A specific VNAR, designated V13, isolated from a *Heterodontus francisci* shark immunized against human vascular endothelial growth factor 165 (VEGF165), exhibited corneal penetration capacity in an animal model without injection or discomfort. This finding underscores the potential applicability of V13 as a novel therapeutic agent for ocular vascular diseases (51).

The combination of small size, stability, and high affinity exhibited by VNARs, together with their demonstrated corneal penetration capability, establishes them as promising tools for developing non-invasive and safer therapies for various ocular conditions, thereby overcoming the limitations of conventional antibody-based treatments.

## 7 Humanization of VNARs for clinical applications

VNARs offer several advantages for use in biomedicine, including their stability, solubility, and small size. However, their small size can be a major disadvantage, as they are quickly removed from systemic circulation through glomerular filtration. For therapeutic purposes, especially those needing extended circulation time, like tumor targeting, it is important to increase their serum half-life (36, 49).

Due to a high sequence similarity between human and camelid VHH domains (approximately 80% homology), humanizing these molecules is relatively easy. These modifications result in biophysically stable, biologically functional, easily expressed VHH domains that maintain almost complete frame identity with human germline sequences (153).

In contrast, humanizing shark VNARs presents a greater challenge due to the structural differences of these molecules compared to the variable domains of human antibodies. VNARs share the classical Ig fold architecture, but their core frameworks are more distantly structurally related to human VH and VL domains (27), T-cell receptor variable domains (154), and other Ig-superfamily cell-surface receptors (155). Because of the evolutionary distance separating sharks and humans, VNARs share little sequence identity with human VH and VL domains (~30% overall) (27), predicting to be the most immunogenic of all the sdAbs.

Therefore, these differences indicate that the humanization of VNAR domains cannot be approached in the same way as the humanization of camelid VHH domains.

In VHHs, the high homology of the framework with human VH3 germline genes and the conservation of the canonical disulfide pattern mean that resurfacing a limited set of solvent-exposed framework residues is often sufficient to obtain human-like domains that preserve their original folding, stability, and paratope architecture (2, 156). In contrast, shark VNAR frames show a much deeper structural divergence, which are noted below: they lack a conventional CDR2 loop; they rely on alternative hypervariable HV2/HV4 regions; and they frequently use lineage-specific non-canonical cysteine ​​patterns to stabilize elongated CDR3 loops, via additional disulfide bonds between CDR3 and FR2/FR4 or between CDR1 and CDR3 (10, 21, 60, 91). The naïve imposition of a human VH/VL-like framework risks disrupting these shark-specific disulfide networks, altering the loop topology and, ultimately, the antigen-binding surface. Structurally, VNAR paratopes are also less segregated from their frameworks than in VHHs. Analyses of antigen-VNAR complexes show that several residues in contact with the antigen reside not only in the CDR1 and CDR3 regions, but also in FR2 and the contiguous HV2–FR3a–HV4 segment, meaning that a substantial fraction of “framework” positions directly shapes the binding surface (49, 157, 158). In parallel, comparative studies of different VNAR isotypes highlight that lineage-specific, non-canonical cysteine patterns in CDR3 and neighboring framework regions (mainly FR2 and FR4) are frequently used to stabilize long, kinked CDR3 loops via additional disulfide bonds, creating shark-specific topologies that have no obvious equivalent in the human germline (10, 13, 154).

However, available crystal structures of VNAR domains demonstrate organization of key FRs like that of human Ig variable domains, thus making humanization possible (27).

The first humanized VNAR was reported by Kovalenko and collaborators, who used the anti-HSA VNAR E06 clone isolated from an immunized spiny dogfish shark as a model to construct humanized variants. Humanization of E06 was carried out by converting more than 60% of non-CDR residues to those of a human germline Vκ1 sequence, specifically DPK9. The resulting huE06 molecules retained the specificity and high binding affinity of the parental VNAR to human, mouse, and rat serum albumins (27).

Despite advances, humanization remains an active area of research. Continued work is necessary to maximize human sequence content in VNARs without compromising binding affinity, stability, and solubility, which could result in immunogenicity due to aggregation.

Concerns persist regarding the potential immunogenicity of VNARs in humans due to their divergent evolutionary origins and low sequence identity with human Igs. Therefore, the next step for promising SARS-CoV-2 neutralizing VNARs would be to address the humanization of their scaffolds (159). **Table 3** compiles representative examples of VNARs selected from different library types and display platforms, highlighting their antigenic targets, potential therapeutic or diagnostic applications, and stage of development.

**Table 3:** Representative VNAR libraries and their applications across display technologies.

SdAbs	Library	Display	Antigenic target	(Potential) Application	Stage of the study	References
SPSL1	Semi-synthetic	Phage displayed	Three toxins; SEB, ricin, and BoNT/A complex toxoid	First demonstration of the utility of a newly created hyper diversified shark VNAR displayed library to serve as a source of thermal stable sdAbs against a variety of toxins.	*In vitro* assays	(50)
SP2, SP6, SP8	Naïve	Phage displayed	CT	Generation of high quality VNAR to agents of interest as required	*In vitro* assays	(98)
DSTL096 and DSTL097	Immunized	Phage displayed	Zaire ebolavirus (used the entire, gamma irradiated virus for shark immunization)	Prophylactic and therapeutic option against Ebolavirus	*In vitro* assays	(92)
H6 VNAR	Naïve	Phage displayed	Precore Hepatitis B e antigen (HBeAg)	Intrabody-based therapy for regulating HBeAg secretion in chronic HBV infection	*In vitro* assays	(178)
IgNAR-653	Synthetic	Phage displayed	Viral hemorrhagic septicemia virus (VHSV)	Neutralizing agent for VHSV in aquaculture disease management	*In vitro* assays	(179)
12Y-2 VNAR	Naïve	Ribosome display	AMA1 from *P. falciparum*	Demonstration of *in vitro* affinity maturation of shark IgNAR using ribosome display; identification of mutations in CDR1, HV4, and CDR3 improving binding	*In vitro* assays	(88)
AC-VNAR1, AC-VNAR2, AM-VNAR1, AM-VNAR2	Semi-synthetic	Yeast surface display	Cetuximab and Matuzumab (anti-EGFR therapeutic antibodies)	Identification of anti-idiotypic VNAR domains targeting monoclonal antibodies (development of specific capturing ligands)	*In vitro* assays	(71)
H8 VNAR	Immunized	Phage displayed	*P. falciparum* histidine-rich protein 2 (PfHRP2)	Improved RDTs for malaria detection	*In vitro* assays	(91)
A tandem multivalent trimer, D1-BA11-C4 and an Fc-fused quadrivalent D1-Fc-C4 (Quad-X™), anti-TNF-α VNAR formats	Immunized	Phage displayed	TNF-α	Showing VNARs as treatment for several inflammatory human diseases dealing with high expression of TNF-α.	Preclinical *in vivo* studies	(180)
3B11, 3B7, 1D11, 2F3	Immunized	Phage displayed	anti-mouse TNF-α (mTNFα)	Development of TNF-α blockers for inflammatory disease therapy and diagnostics	*In vitro* assays	(181)
B2 VNAR	Semi-synthetic	Phage displayed	PD-L1	Development of CAR-T therapies targeting PD-L1 for breast and liver cancer treatment	*In vivo* assays (xenograft mouse models)	(140)
Vnarbody 20G6 and 17F6	Immunized	Phage displayed	RBD domain of SARS-CoV-2	prophylactic and therapeutic option against most SARS-CoV-2 variants	*In vivo* assays	(182)
ShAb01, ShAb02, BiShAb0201	Immunized	Phage displayed	SARS-CoV-2 RBD and related sarbecoviruses	Neutralization of SARS-CoV-2 variants and prophylactic protection against sarbecoviruses	*In vivo* assays	(183)
vNAR T1	Naïve	Phage displayed	Transforming growth factor beta (TGF-β) isoforms; 1, 2 & 3	Modulation of TGF-β levels implicated in several human diseases such as fibrosis, cancer, and COVID-19.	*In silico* assays	(184)
biNb-2A2 and biNb-3D	Immunized	Phage displayed	HpA adhesin of *Helicobacter pylori*	treatment or diagnostics of *H. pylori* infection	*In vitro* assays	(185)
D02 vNAR	Immunized	Phage displayed	ORF064L viral protein in Decapod iridescent virus 1 (DIV-1)	development of VNAR for DIV-1 detection in shrimp culture	*In vitro* assays	(186)
VNAR1, VNAR11, VNAR21, VNAR25	Immunized	Phage displayed	α-Fetoprotein	Serum biomarker diagnosis for primary liver cancer	*In vitro* assays	(81)
TXB2	Semi-synthetic	Phage displayed	TfR1	BBB transport; CNS delivery of therapeutic proteins and antibodies	*In vivo* assays	(37)
F9, F11, G3	Immunized	Phage displayed	CD-20 antigen	Therapy against lymphoblastoma by inhibiting the action of the CD-40 antigen	*In vitro* assays	(81)
VNAR-5G8	Immunized	Phage displayed	human trophoblast cell surface antigen 2 (TROP-2)	Immunotoxin with strong affinity to TROP-2. Antitumor activity	*In vitro* and *in vivo*	(165)
AuNPs@PSV	Naïve	Phage displayed	Crustacean Tropomyosin (TM)	Screening of crustacean TM in food (allergy)	Generation of a lateral flow immunochromatographic assay (LFIA)	(84)
H4, H15, H17, NGS2405, Fc conjugated	Immunized	Phage displayed	Fibroblast activation protein (FAP)	Development of therapies to dampen immunosuppression	*In vitro* and *in vivo*	(164)

In summary, humanization of VNARs is a key process for translating their therapeutic promise from the laboratory to the clinic. By engineering their sequence and evaluating their biophysical and immunogenic properties, the goal is to develop a new class of safer and more effective biological agents for a wide range of diseases.

## 8 Conclusions

VNARs, single-domain Abs derived from sharks, represent a disruptive innovation in medical biotechnology by offering unique structural and functional properties compared to conventional Abs. Their small size, high solubility, and remarkable thermostability provide significant advantages in diagnostic and therapeutic applications, especially in situations that require tissue penetration, resistance to extreme conditions, or access to hidden epitopes. The diversity of selection platforms, along with the development of immune, naïve, and synthetic libraries, has expanded their repertoire and adaptability, making them versatile tools in immunodiagnostics, oncology, infectious diseases, and autoimmune disorders.

However, challenges remain for their full clinical implementation, particularly the need to humanize them and extend their half-life in circulation — key aspects that ensure safety and efficacy in advanced therapies.

Academic centers, such as the University of Aberdeen, have become a leading center for VNAR research, notably in advancing therapies with humanized shark Abs, such as the BA11 clone. Elasmogen, an Aberdeen-based biopharmaceutical company, leverages its soloMER™ platform — humanized single-domain Abs derived from sharks — to develop stable, versatile biologics that can target challenging environments, such as the eye and intestine. Their intellectual property covers all aspects of soloMER generation and application, enabling broad clinical translation (160). With partners like Almac Discovery, Elasmogen is advancing ALM-401, a bispecific ADC targeting epidermal growth factor receptor (EGFR) and receptor tyrosine kinase-like orphan receptor 1 (ROR1) in oncology and is also pursuing topical treatments for ocular diseases and oral therapies for inflammatory bowel disease via partnerships with Intract Pharma. The company’s pipeline further includes candidates for autoimmune and intestinal inflammatory disorders (161, 162).

This candidate is half the size of conventional ADCs, favoring manufacturing and tumor access. Beyond cancer, Elasmogen is developing innovative treatments for ocular inflammatory diseases, aiming to replace corticosteroids and injectable biologics with stable, soluble, topically delivered soloMER drops. In inflammatory bowel disease, a strategic alliance with Intract Pharma is enabling oral delivery of anti-inflammatory soloMERs using Soteria^®^ and Phloral^®^ technologies for targeted colonic release, maximizing therapeutic outcomes, and minimizing off-target side effects. The company also maintains a pipeline addressing systemic autoimmune and intestinal inflammatory disorders, supported by public and private investment. Collectively, Elasmogen’s portfolio highlights the versatility of soloMERs as a revolutionary therapeutic scaffold with applications spanning oncology, ophthalmology, gastroenterology, and immunology, underscoring their potential to address unmet clinical needs with site-specific, cost-effective, and next-generation biologics (163). Other notable VNAR advances stem from the National Institutes of Health (NIH) (164) and Chinese institutions, such as Jimei University, which are expanding the therapeutic repertoire (81, 165).

On the other hand, Ossianix specializes in CNS applications by developing VNAR-based brain shuttles for drug delivery across the blood-brain barrier, collaborating with Lundbeck. As the biopharmaceutical industry adopts next-generation Abs, VNARs are gaining increasing value for their stability, manufacturing efficiency, and precise targeting capabilities. Current preclinical efforts focus on applications ranging from diagnostics to innovative therapies, particularly in oncology and other areas of unmet medical need (166).

In conclusion, VNARs are projected as a promising platform capable of transforming the diagnosis and treatment of multiple diseases, thereby consolidating their place in the new era of medical biotechnology.
